# Supplementary material for: Detonation synthesis of carbon nano-onions via liquid carbon condensation
Source: Nat Commun. 2019 Aug 23;10:3819. doi: 10.1038/s41467-019-11666-z (PMC6707243; doi:10.1038/s41467-019-11666-z)
Supplement: Supplementary file 1 — Supplementary Information [file 41467_2019_11666_MOESM1_ESM.pdf]

Supplementary Information

Detonation synthesis of carbon nano-onions via liquid carbon condensation

Bagge-Hansen *et al.*

* Corresponding author. Tel: (925) 424-6084. E-mail: willey1@llnl.gov (Dr. Trevor M. Willey)

Supplementary Methods

TR-SAXS data obtained during the detonation of DNTF reveal subtle changes during detonation that suggest excess carbon forms liquid phase nano-droplets that freeze into solid, graphitic carbon nano-onions over the first ~200 ns post-detonation. In this section, the underlying SAXS theory, detailed analysis, and degree of certainty are presented. The goal of the SAXS modeling is to fit all the SAXS data to the same model with as few fit parameters and assumptions as possible. The model should account for a size distribution with mean radius, $\bar{R}$, and standard deviation, σ, and intra-particle heterogeneity, $\Delta\rho$. In addition to these three parameters, a scaling factor, K_{onion}, and flat background, b, are included to account for any change in the volume fraction of the onions and high-q gas scattering, respectively. In total, these five parameters can be reliably extracted from each SAXS data set and are discussed in detail below.

TEM imaging observations of recovered detonation products further frame and inform our analysis. Figure 4a shows a representative TEM micrograph at high magnification from post-detonation products collected from DNTF. Complementary low magnification TEM micrographs are shown in Supplementary Figure 1. The microscopy reveals an abundance of graphitic spherical nanoparticles, or nano-onions. These particles consist of concentric graphitic spherical shells, with overall particle diameters of ~10 nm and interlayer separation of 3.1 Å. These TEM morphology data provide foundational input to construct a physical model that we employ to fit the SAXS data obtained at all times after the detonation front passes through the X-ray beam.

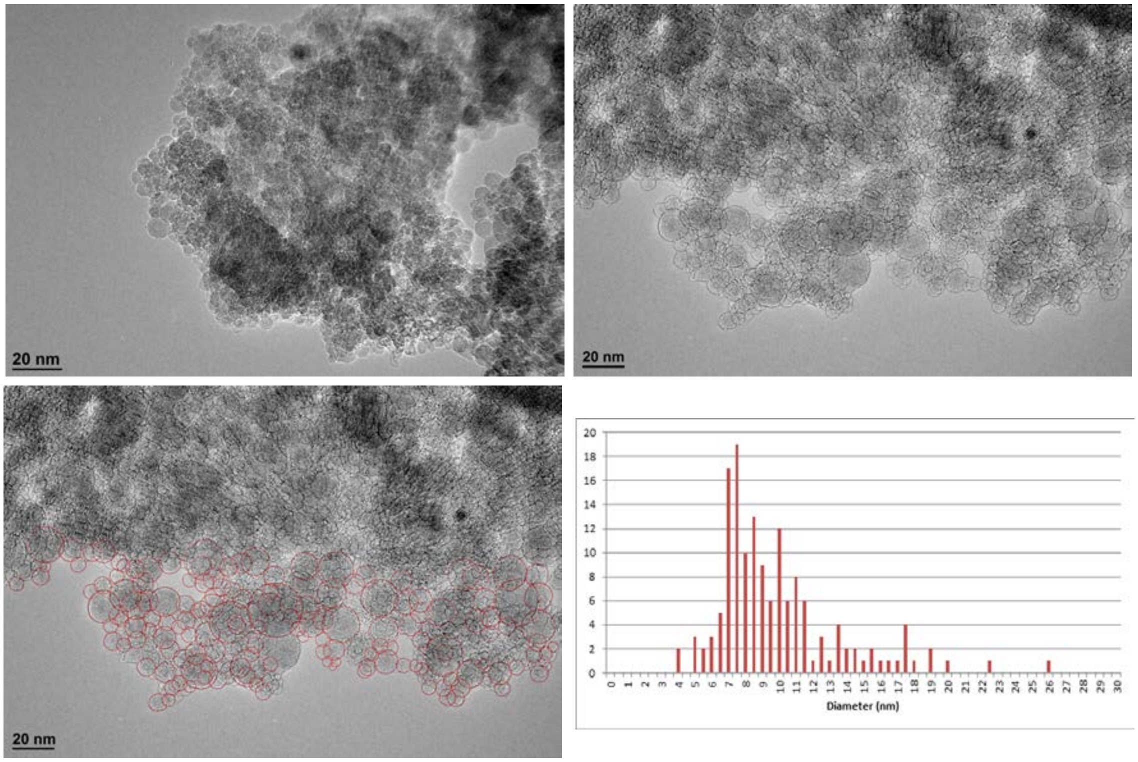

Supplementary Figure 1. Representative TEM micrographs at low magnification demonstrate the abundance and consistency of the nano-onions in the detonation products of DNTF. A size distribution is also extracted from these data for direct comparison to the SAXS data (see below).

Carbon nano-onions (hereafter onions) are spherical and have evenly separated concentric layers. We take advantage of this spherical symmetry[1] to model the onions as concentric core-shells. For a scattering object with spherical symmetry phase, the scattering intensity, $I\,(q)$, depends on the radial scattering length density function, $\rho(r)$, following:

(1)
$$I(q) = \left[4\pi \int_0^R \rho(r) \frac{\sin qr}{qr} r^2 dr\right]^2$$

where the integral extends out to the radius of the particle, R.[1] For a homogeneous function, $\rho(r)$, the integral inside of Supplementary Equation 1 (with the factor of 4π) has an exact solution that is colloquially known as the scattering amplitude of a sphere, $A_s(R, q, \Delta\rho)$. [1] For an onion, the function, $\rho_{onion}(r)$, is periodic, with a spacing of 3.1 Å, as the electron density is highest near the sp_2 carbon concentric shells and lowest in between adjacent shells (Supplementary Figure 2). The discretized form of Supplementary Equation 1 has been implemented in many different, core-shell, systems by: 1) replacing the continuous function, $\rho(r)$, with discrete Heaviside functions, 2) partitioning the limits of the definite integral across each step and 3) using the integration by parts approach to solve the definite integral across each step. Using this formulation, the scattering from concentric carbon layers can be obtained by approximating the otherwise continuous function ρ as a series of Heaviside functions shown in Supplementary Figure 2. For an onion-like particle that contains carbon concentric shells, the Heaviside functions in Supplementary Figure 2 continue to

alternate until the overall radius R of the particle is exceeded. Therefore, the model function, $\rho_{onion}(r)$, used here begins with an electron depleted region and alternates until R would be exceed, which means that the minimum size difference between onion particles of different size is 3.1 Å. The electron density of the core region of ρ_{onion} ($0 > r > l$) is assumed to be the same as the depleted electron region for simplicity, since its exact size and density is a small contribution to the scattered intensity. Beyond the onion particle, the scattering length density of the bulk is approximated as undetonated DNTF, ρ_{DNTF}, since the mass has not expanded appreciably at early times that are important in this study. Performing this integral on Supplementary Equation 1, and normalizing to the volume of the particle one obtains:

$$I_{onion}(q,R)=\frac{\left[\left[\sum_{r_N=l}^{r_N=R-l} A_s(r_N,q,[\rho_d\text{-}\rho_c])\right]+\left[\sum_{r_N=l}^{r_N=R-3l} A_s(r_N+l,q,[\rho_c\text{-}\rho_d])\right]+A_s(R,q,[\rho_c\text{-}\rho_{DNTF}])\right]^2}{V(R)} \quad (2)$$

where, l represents the distance over steps where $\rho_{onion}(r)$ is held constant (half the graphitic layer to layer distance), ρ_c is the scattering length density of the electron-rich graphitic layer, ρ_d is the scattering length density of the electron-poor region between the graphitic layers, and the summations begin at l and increment by $2l$. The first summation accounts for the electron-poor shells and the second summation accounts for electron-rich shells; the summation is split into two parts to emphasize that the contrast of the last layer contains the scattering length density outside the onion, ρ_{DNTF}. In Supplementary Equation 2, the function, $A_s(r,q,[\rho_c\text{-}\rho_d])$ is the well-known q-dependent scattering amplitude of a sphere with radius, r, and contrast, $\rho_c\text{-}\rho_d$ and $V(R)$ is the volume of a sphere. A value of 25 x 10^{10} cm^{-2} was assumed for ρ_c, which is very close to what one would expect for the average electron density in the vicinity of the carbon nuclei (Supplementary Figure 2). Slightly larger or smaller values could also be assumed, and would affect only the scaling of the contrast, $\Delta\rho$. Practically, within the scope of small angle scattering theory, the scattering phase must be much larger than the wavelength of the X-rays and therefore neither the magnitude of l nor ρ_c can be uniquely extracted from the SAXS data. Therefore, we use the theoretical value of 1.55 Å for l and an estimated value of ρ_c (Supplementary Figure 2). By fixing these values, we are able to reduce the number of fit parameters to determine whether the there is intra-particle heterogeneity at each point in time. This is because only the interference scattering[2] between layers, within the onion, can be observed in our SAXS data and shown in Supplementary Figure 3.

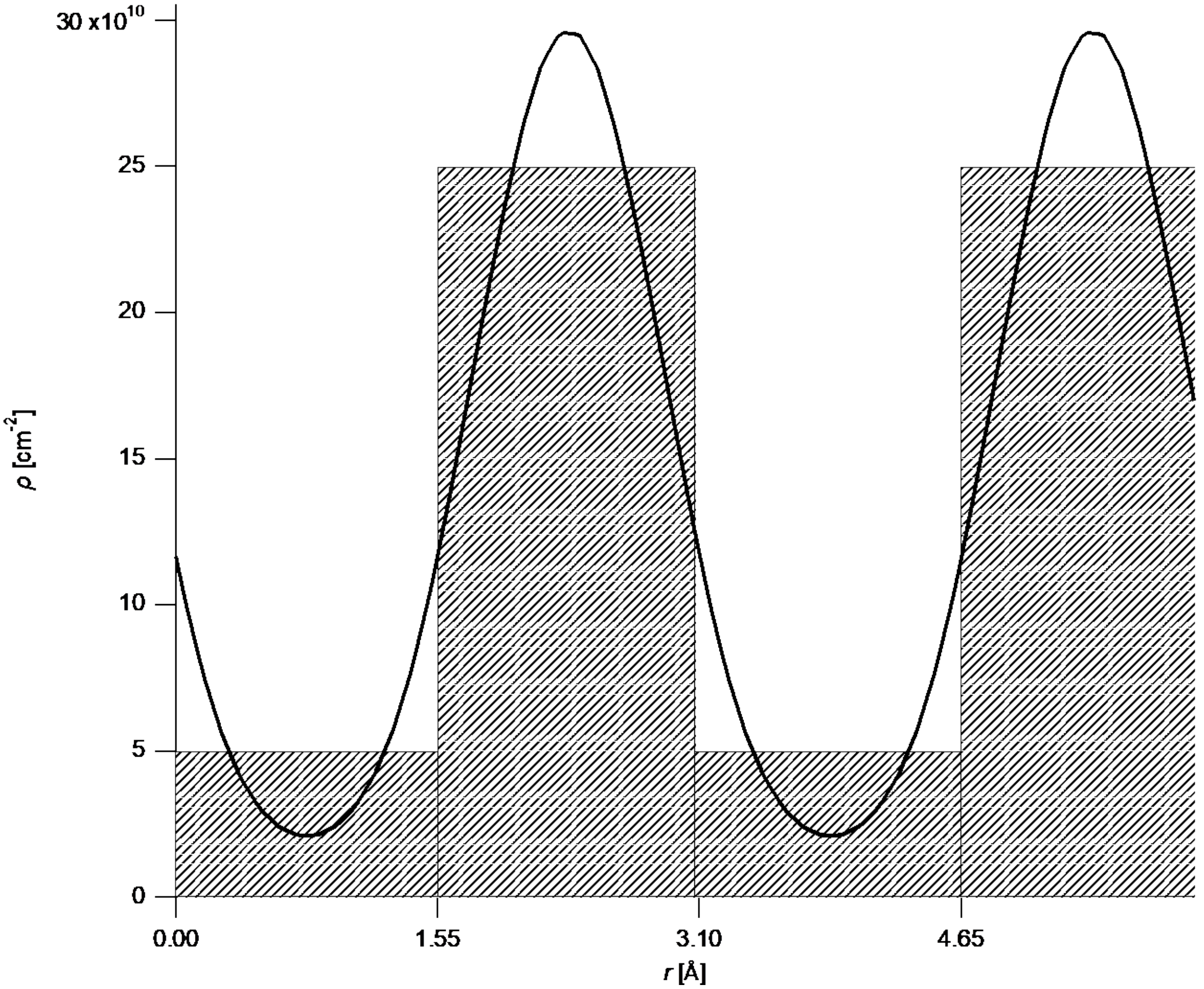

Supplementary Figure 2. Absolute scattering length density, *ρ*, which is expected from adjacent graphene layers (black line), calculated using density functional theory, and a series of Heaviside functions that represent the periodic scattering length density and are used to model the SAXS data.

Using Supplementary Equation 2, the SAXS obtained from the detonation products, $I_{dp}(q)$, can be modeled as a size distribution of either heterogeneous onions or homogenous particles. The shape of the size-distribution was assumed to be a log-normal volume distribution, $D_{\ln}(R, \bar{R}, \sigma)$, which is a well-used distribution in small angle scattering:

(3)
$$D_{\ln}(R, \bar{R}, \sigma) = \frac{1}{\sigma R\sqrt{2\pi}} \mathrm{e}^{\left[\frac{-\left[\ln\frac{R}{\bar{R}}\right]^2}{2\sigma^2}\right]}$$

where $\bar{R}$ is the median radius and σ is the standard deviation. For an onion, only discrete values of R are allowed, which are determined by l. This discretization does not significantly affect the scattered intensity, since we cannot resolve particle size differences of 3 Å and less. The discretized size distributions are

shown in this document. The scattered intensity from the detonation products, $I_{dp}(q)$, is modeled by the equations:

(4)
$$I_{\mathrm{dp}}(q) = \frac{K_{\mathrm{onion}}}{\langle \Delta\eta \rangle^2} \sum_0^R I_{\mathrm{onion}}(q) D_{\mathrm{ln}}(R, \bar{R}, \sigma) \Delta R + b$$

(5)
$$\langle \Delta\eta \rangle^2 = \left[\left[\frac{\bar{R} - l}{\bar{R}} \right]^3 [\rho_{\mathrm{c}} - \rho_{\mathrm{DNTF}}] + \sum_{r_{\mathrm{N}}=l}^{r_{\mathrm{N}}=R-l} \left[\left[\frac{r_{\mathrm{N}}}{\bar{R}} \right]^3 [\rho_d - \rho_c] + \left[\frac{r_{\mathrm{N}} + l}{\bar{R}} \right]^3 [\rho_{\mathrm{c}} - \rho_{\mathrm{d}}] \right] \right]^2$$

where, K_{onion} is a scaling constant, and $\langle \Delta\eta \rangle$ is a volume-average-normalization factor that is necessary to reduce the coupling of ρ_{d} and K_{onion} in Supplementary Equation 4. Altogether, there are five parameters to fit to the SAXS data: b, $\bar{R}$, σ, K_{onion} and ρ_{d}. Supplementary Equation 4 is parameterized in such a way that the layer contrast, $\Delta\rho = \rho_{\mathrm{c}} - \rho_{\mathrm{d}}$ is fit to the data and increases as the onion layers becomes more discrete, e.g., during freezing, and is shown schematically in Supplementary Figure 3. While Supplementary Equation 4 can distinguish between a homogenous and heterogeneous sphere, it is not possible to resolve the morphological detail during the transition between the two states illustrated in Supplementary Figure 3; for example, it is not possible to determine between a half-core, half-onion state and an onion, which is uniformly expanding.

abbreviation	description	<0.1 μs	> 0.4 μs
K_{onion}	Scaling constant that is proportional to the volume fraction at early times when ρ_{DNTF} is valid prior to gas expansion. Affects intensity of scattering.	0.36 cm^{-4}	1.17 cm^{-4}
ρ_d (Δρ)	Average scattering length density of the region between carbon layers within the onion. (the contrast between electron-rich, ρ_c, and electron poor regions, ρ_d) ρ_c - ρ_d is depicted as Δρ and, represents how far graphitization has progressed. It is this parameter that is plotted against computed phase (liquid-graphite) with time in the main text.	25 x 10^{10} cm^{-2} (0 cm^{-2})	5.7 x 10^{10} cm^{-2} (19.3 x 10^{10} cm^{-2})
$\bar{R}$	Median particle radius	45 Å	48 Å
σ	Normalized standard deviation of the log-normal distribution	0.38	0.42
b	Flat background scattering, which is mostly dominated by elastic gas scattering and incoherent scattering.	<0.001 cm $^{-1}$	0.024 cm $^{-1}$

Supplementary Table 1. Abbreviations, descriptions, and values of each parameter varied in Supplementary Equation 4. The values shown before and after complete freezing and graphitization are shown in the columns labeled < 0.1 μs and > 0.4 μs, respectively.

abbreviation	description	Value
ρ_c	Estimated scattering length density within 0.775 A of either side of the sp2 carbon plane.	25 x 10^{10} cm^{-2}
ρ_{DNTF}	Calculated scattering length density of the surrounding gas based on the density and composition of the DNTF pellet. This approximation is valid when the detonation products have not appreciably expanded beyond the original shape of the pellet early in the detonation.	14.5 x 10^{10} cm^{-2}
l	This is the radial thickness of the electron-rich and electron-poor regions of a graphitic-onion that has periodicity of 3.1 Å between carbon planes as measured by electron diffraction in the TEM.	1.55 Å
$A_s(R, q, \Delta\rho)$	This is the scattering amplitude of a sphere that is obtained from the integration of Supplementary Equation 1.[2]	$A(R,q,\Delta\rho) = V(R)\Delta\rho \frac{3[\sin qR - qR\cos qR]}{[qR]^3}$

Supplementary Table 2. Abbreviations, descriptions, and values of each parameter used in the SAXS data modeling.

Phase changes can be revealed by extracting $\Delta\rho$ as a function of time, immediately post-detonation (Figure 2b), to determine when the onion-like particles are observed. However, the effect of $\Delta\rho$ on the SAXS model is highly non-linear and, for small values, has a similar effect on the scattering shape as the flat background, b, and the width of the distribution, σ, at high-q and low-q, respectively. Therefore a threshold value of $\Delta\rho$,

above which we consider the particles to be heterogeneous, and below which we consider the particles to be homogenous. In order to establish this threshold, a broad size distribution of particles is assumed (σ = 0.5) and an average background value; both of these conditions require a higher $\Delta\rho$ than narrow size distributions and lower backgrounds, which are often observed at detonation times less than 0.2 μs. From Supplementary Figure 3, we observe that for values of $\Delta\rho$ between 0 and 10 x 10^{10} cm^{-2} has very little effect on the scattered intensity. On the other hand, the scattering from particles with values of $\Delta\rho$ greater than 18 x 10^{10} cm^{-2} is significantly different. Therefore, the scattering from particles with $\Delta\rho$ ~ 15 x 10^{10} cm^{-2} are considered to have observable heterogeneity and scattering from particles with $\Delta\rho$ > 18 x 10^{10} are considered to have clear heterogeneity. The error bars for $\Delta\rho$ shown in the main article were taken from the lmfit package, except for values near 15 x 10^{10} cm^{-2}, where the maximum value from either the global chi-square analysis or 3 x 10^{10} cm^{-2} was used.

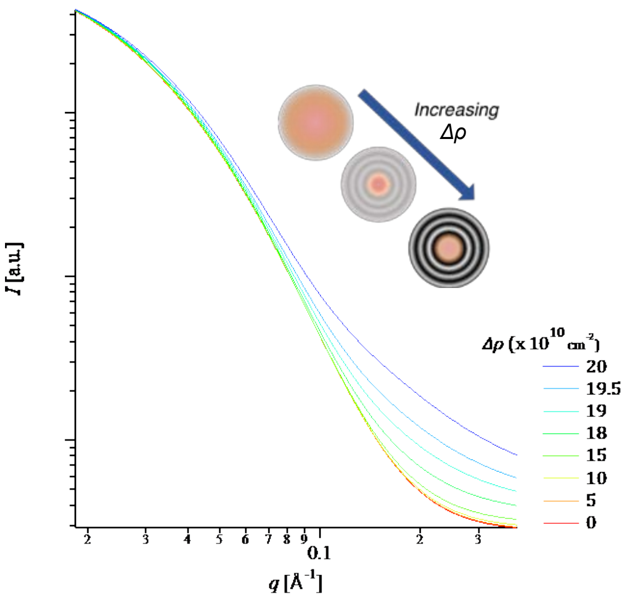

Supplementary Figure 3. Theoretical scattering from an onion with varying degrees of separation in the electron contrast, *Δρ*, between layers.

The SAXS data collected from a total of ten different DNTF shots was fit using Supplementary Equation 4 for the model intensity, $I_m(q)$, using the least-squares fitting available in the lmfit package[3] for python. The model parameters and standard errors were extracted and presented below. The values of $\Delta\rho$ obtained from each fit are shown in Figure 2b. Based on the model fitting, and as discussed in the main paper, the transition from uniform sphere to onion occurs within 0.2 μs ± 0.1 μs, reaching a value greater than 18 x 10^{10} cm^{-2} and is of sufficient magnitude to warrant confidence that the trend is significant (see above).

The total volume fraction of detonation products can also be estimated from Supplementary Equation 4 by the relation:

(6) $$\nu_{\text{onions}} = \frac{K_{\text{onion}}}{\langle \Delta\eta \rangle^2}$$

where all terms are defined previously. In general, there are large errors in ν_{onions} since the uncertainty includes the uncertainty in the layer contrast, $\Delta\rho$, and K_{onion}. The values of ν_{onions} are considered maximum values, since the scattering length density is expected to dramatically decrease after the detonation front passes. Using Supplementary Equation 6 and assuming a constant transmission, X-ray path length and scattering length density of the bulk, we estimate that the onions occupy less than 1 % of the volume fraction within 0.3 μs after the detonation wave passes. This number is very close to 2 %, which is estimated from thermochemical simulation.

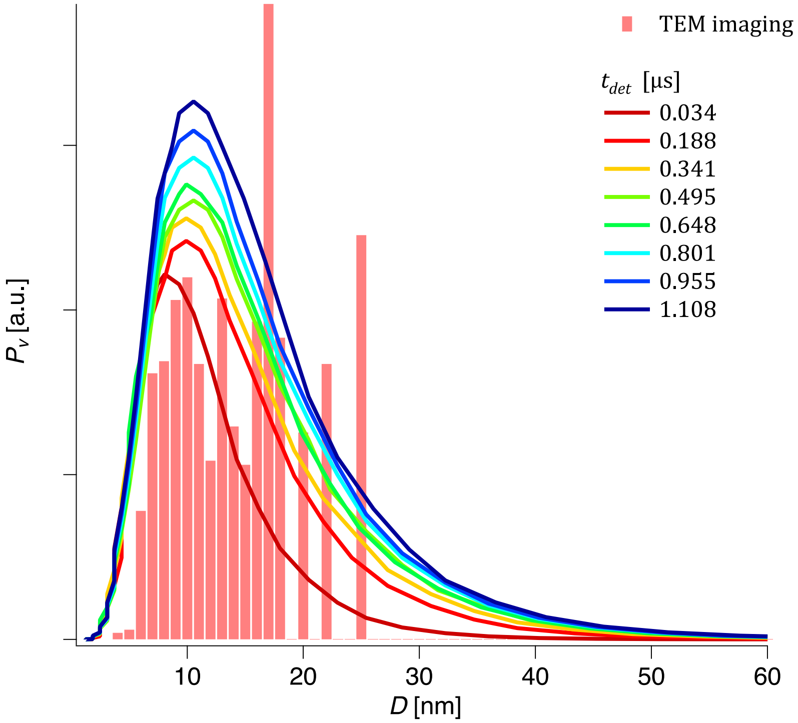

Supplementary Figure 4. Comparison of the volume fraction distributions, P_v, obtained from SAXS data (single shot of campaign 2) and TEM imaging.

The size distribution of onions is also obtained from the model fitting and can be compared directly with the size distribution from TEM imaging (Supplementary Figure 1) to validate the TR-SAXS data modeling. However, there is large difference in the number of particles measured with a beam size of ~ 0.08 mm x 0.08 mm and a pellet diameter of 6 mm, compared with TEM imaging. For example, the total number of 10 nm diameter particles that occupy 1 % of the volume in is on the order of 10^{11} particles, as measured by TR-SAXS. Because far fewer particles were counted from the TEM imaging, the relative volume fractions from each technique are used for comparison. The time-resolved evolution in the onion volume distribution extracted from the SAXS data (Figure 2a) is shown in Supplementary Figure 4 with the equivalent volume distribution histogram, $P_v(D)$, obtained from TEM data by multiplying the number frequency histogram $P_N(D)$

by the volume, $V(R)$. The equivalent volume fraction histogram is obtained by integrating $D_{\ln}(R, \bar{R}, \sigma)$ across each bin, ΔR. In general, there is good agreement between the size distribution at late times and that obtained from TEM imaging. There appears to be a higher volume fraction of 17 nm diameter particles observed by TEM imaging. However, these particles are a small fraction of the total number counted and are also near the upper limit of sizes that can be resolved from the measured q-range[2] ($2\pi/q_{min}$ = 30 nm). Within the scope of this study, it is important that the particle distribution for smaller sizes is accurately modeled, as it the smaller particle sizes that contribute in a similar way as the intra-particle heterogeneity at high-*q.* Based on the comparison shown in Supplementary Figure 4, we conclude that the sizes obtained from the TR-SAXS modeling does agree well, which is confirmed by TEM imaging and shown in Supplementary Figure 4.

All of the SAXS data and model fits to the onion are shown in Supplementary Figures 5 and 6 for the 1st and 2nd experimental campaigns, respectively.

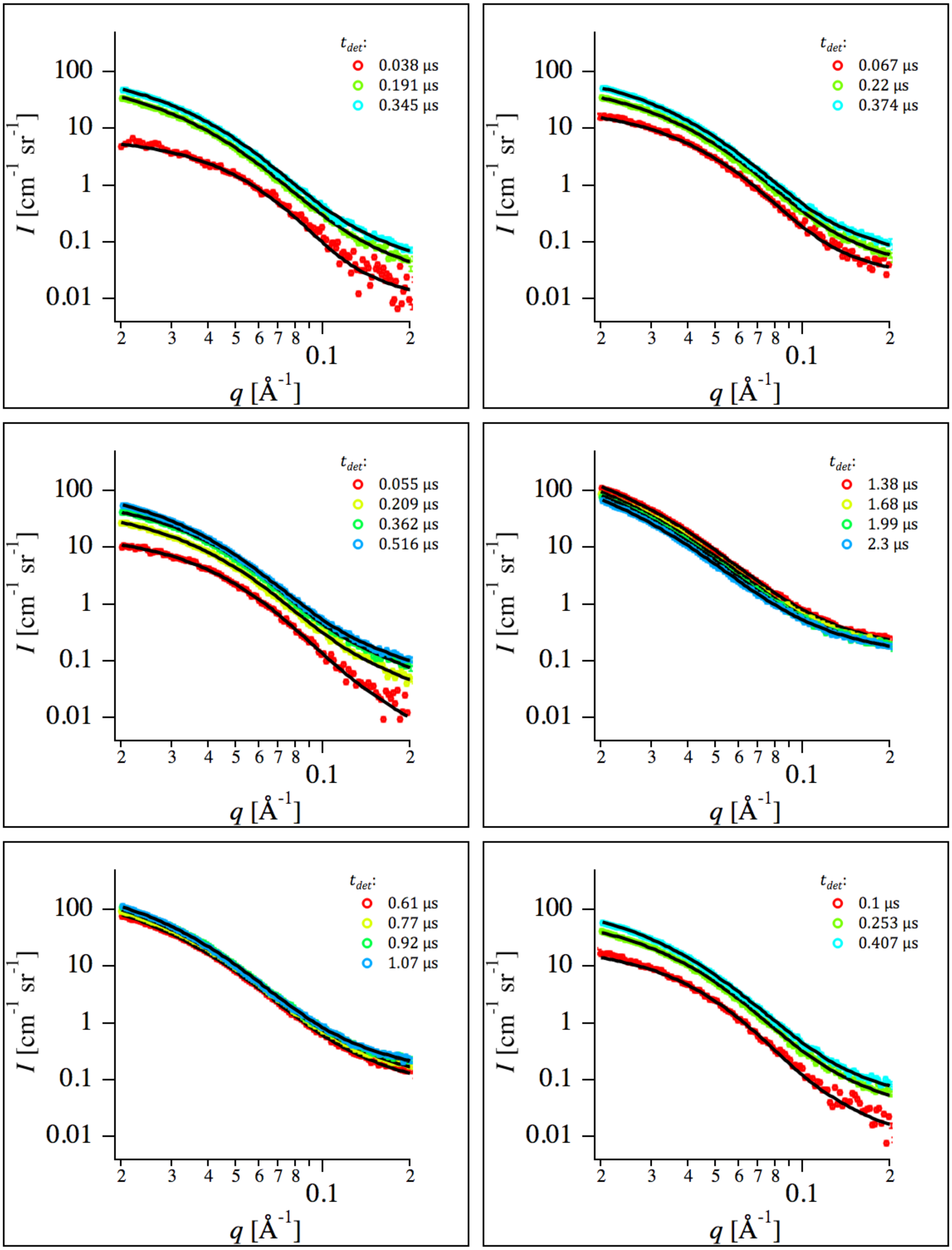

Supplementary Figure 5. TR-SAX data collected from campaign 1 with direct comparison to model fits (Supplementary Equation 4; onion model).

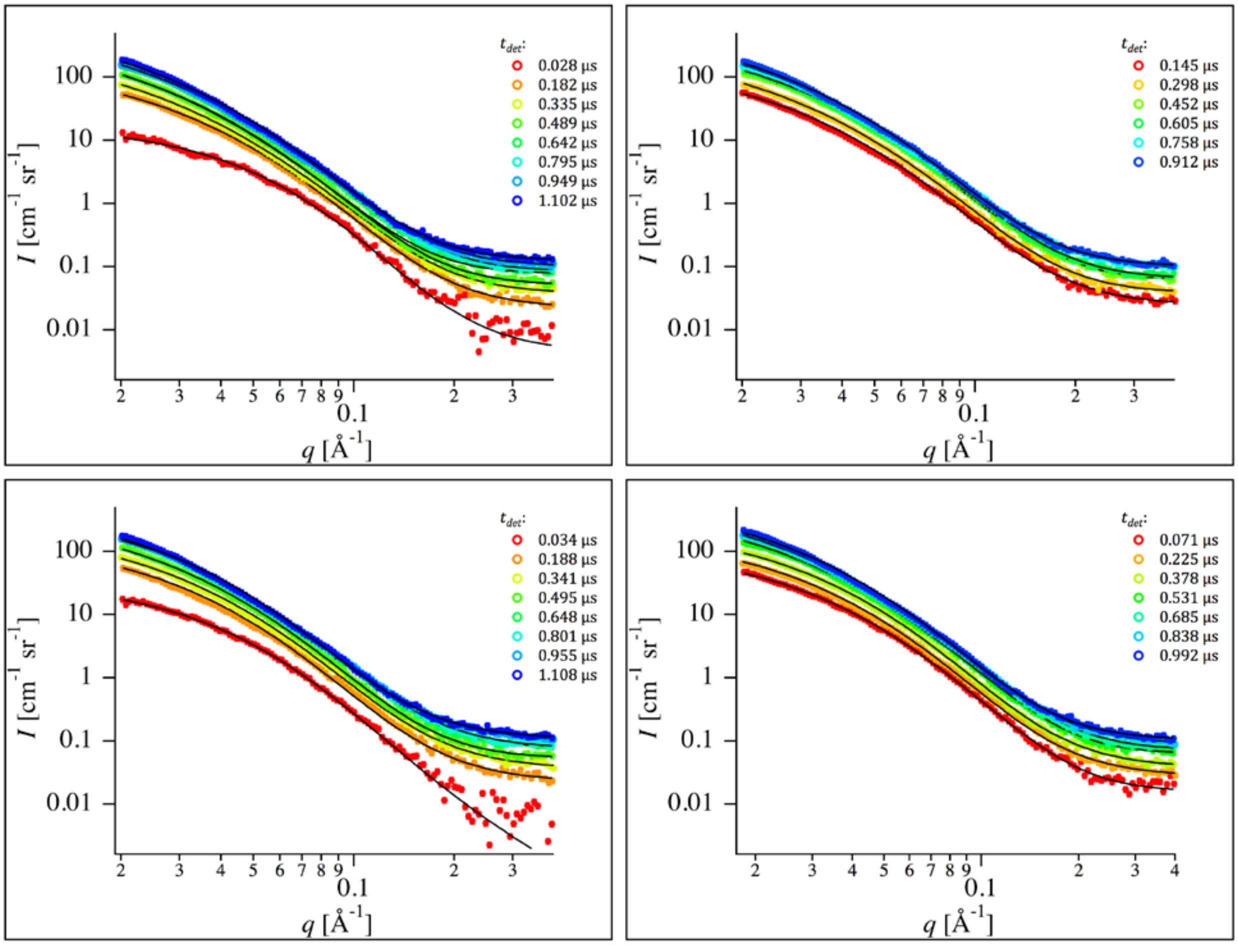

Supplementary Figure 6. TR-SAX data collected from campaign 2 with direct comparison to model fits (Supplementary Equation 4; onion model).

The small angle X-ray scattering data reported in the main article is non-traditional, compared with the SAXS data normally reported in literature. In an ideal SAXS experiment, the X-rays are monochromatic, which makes the wave transfer vector, **|q|**, only a function of the scattering angle, θ by the equation:

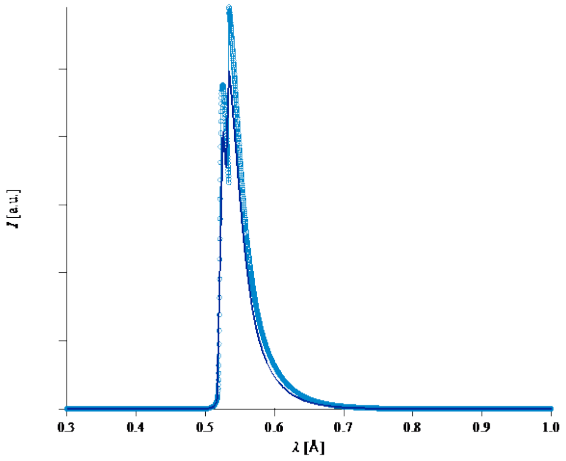

Supplementary Figure 7. Simulated plot of the U18 undulator profile, U(λ), (circles) with the corresponding transmission function, *T*(λ), (line). The function, *T*(λ), is calculated for 5 mm AgBeh.

(7) $$q = \frac{4\pi \sin\theta/2}{\lambda}$$

where λ is the wavelength of the X-rays. For synchrotron X-rays, a monochromator is typically used to only allow a very narrow distribution of wavelengths to pass through the sample and thereby reducing the incident intensity. In most cases, the intensity loss is of very little consequence and can be compensated by increasing the exposure time to seconds or minutes; however, such long exposure times were not possible for our experiments because of the short time scales of post-detonation carbon condensation. Therefore, no monochromator was used and the incident X-rays had a wavelength distribution, *U*(*λ*), owing to the undulator profile. As the function, *U*(*λ*), is known (Supplementary Figure 7), this correction can be made and is presented here.

The wavelength smearing is accounted for by scaling the discrete scattered intensity of each wavelength in *U*(*dλ*) according to the wavelength-dependent properties of the sample and scintillator. The subsequent scaled function, *R*(*λ*), is used as a weighting function to scale the monochromatic scattered intensity, *I*(*q*), and integrated over the *q*-range defined by the monochromatic *q* value and the minimum and maximum wavelengths in *U*(*λ*). The smeared intensity, $I_{\mathrm{sm}}(\langle q\rangle)$, is therefore given by the equations:

(8) $$I_{\mathrm{sm}}(\langle q\rangle) = \int_{q_{\min}}^{q_{\max}} R(q,\langle q\rangle) I_{\mathrm{m}}(q)\, dq$$

(9) $$R(q,\langle q\rangle) = \frac{T\left(\langle\lambda\rangle \frac{q}{\langle q\rangle}\right)\rho^2\left(\langle\lambda\rangle \frac{q}{\langle q\rangle}\right)\eta_{\mathrm{sc}}\left(\langle\lambda\rangle \frac{q}{\langle q\rangle}\right)}{\int T\left(\langle\lambda\rangle \frac{q}{\langle q\rangle}\right)\rho^2\left(\langle\lambda\rangle \frac{q}{\langle q\rangle}\right)\eta_{\mathrm{sc}}\left(\langle\lambda\rangle \frac{q}{\langle q\rangle}\right) dq}$$

(10) $$T(\lambda) = U(\lambda)\mathrm{e}^{\left(-{}^{\mu}/_{\rho(\lambda)}\, dt\right)}$$

Where $q_{\min}$ and $q_{\max}$ are obtained from $\lambda_{\min}$ and $\lambda_{\max}$ in Supplementary Figure 7 and applying Supplementary Equation 9, *T*(*λ*) and $\rho^2(\lambda)$ are the wavelength-dependent transmission and scattering signal for the sample, respectively, and $\eta_{sc}(\lambda)$ is the wavelength-dependent scintillator efficiency and $\langle\lambda\rangle$ is chosen so that the peak positions from the theoretical polychromatic silver behenate scattering standard are the same as monochromatic scattering. The dominant function in Supplementary Equation 9 is the transmission. The scattering length density, *ρ*, is also a function of *λ* but does not vary significantly for the HE material; however, $\rho^2(\lambda)$ does vary for silver behenate since Ag has an absorption edge at ~ 24 keV. Therefore, $\rho^2(\lambda)$, is only included in the theoretical wavelength smearing calculated from monochromatic Ag Behenate. The scintillator efficiency, $\eta_{sc}(\lambda)$, for LSO is non-linear in the X-ray energy range: 10 keV to 100 keV and varies between 0.55 and 1.0;[4] in these prior works[4], thicker single crystals were used, while thin LSO powder films were used here. Considering the undulator profiles in Supplementary Figure 7, $\eta_{sc}(\lambda)$ varies less than 10%, which is far less than *U*(*λ*). Nevertheless, $\eta_{sc}(\lambda)$ is included for completeness. The smearing function, $R(q,\langle q\rangle)$, for silver behenate is shown in Supplementary Figure 6 and is very similar to *U*(*λ*). Finally, the function, $R(q,\langle q\rangle)$, must be normalized such that $I_{\mathrm{sm}}(\langle q\rangle)$ is scaled correctly. Overall, the wavelength smearing is verified by comparing the theoretical and measured polychromatic scattered intensity curves,

$I(q)$, from Ag behenate and glassy carbon. In general, this affect is near negligible for the onion model, using $U(\lambda)$ in Supplementary Figure 6, and does not significantly change the modeling results.

The exposure times that are a factor of 10^3 less than what is commonly used in time resolved SAXS measurements were made possible only by increasing the signal to noise via two image intensifiers (micro-channel plate from Photek and PI-MAX4 cameras) and by using a pink beam rather than monochromatic X-rays. This monumental leap in time-resolution requires some consideration in the data reduction and analysis, which are presented in detail in this section. Specifically, the wavelength smearing (previous section) and background subtraction from partially detonated and fully detonated SAXS data are presented and discussed. A detailed discussion of the micro-channel plate can be found in our prior publication[5]. We note that the gain for the micro-channel plate was set to minimize saturation at low-*q*, maintain intensity/count linearity while increasing the signal to noise at high q where the intensity is lowest. The raw images obtained from the first campaign (Figure 2a) are provided along with dark field, empty field (background) and Ag behenate in Supplementary Figure 8.

The small angle scattering from the phase of interest (here, carbon condensates) must be separated from all other scattering phases (i.e., background). This mathematical operation is accomplished by collecting the SAXS from the background, $I_b(q)$, and subtracting it from raw intensity, $I_{raw}(q)$, to obtain the intensity of the detonation products, $I_{dp}(q)$.

(11)
$$I_{\mathrm{dp}}(q) = \frac{C}{t}\left[\frac{I_{\mathrm{raw}}(q)}{\mathrm{Tr}} - I_{\mathrm{b}}(q)\right]$$

where Tr is the transmission of the detonation products, *C* is a calibration factor and *t* is the path-length of the X-rays through the detonation products. Oftentimes, the incident and transmitted intensity are measured simultaneously with $I_b(q)$ and $I_{raw}(q)$ and subsequently used to calculate the transmission, Tr. However, this was not possible for our experiments. Instead, Tr was taken to be a minimum value, Tr^*, that corresponds to the static HE sample. From Supplementary Equation 11, a minimum transmission, Tr^*, guarantees positive values of $I_{dp}(q)$ and is a good approximation at the early detonation times. However, the uncertainty in Tr is evaluated and discussed below. One can expect the real value of Tr to vary between that of the static sample, Tr* (0.73 for DNTF), and 1.

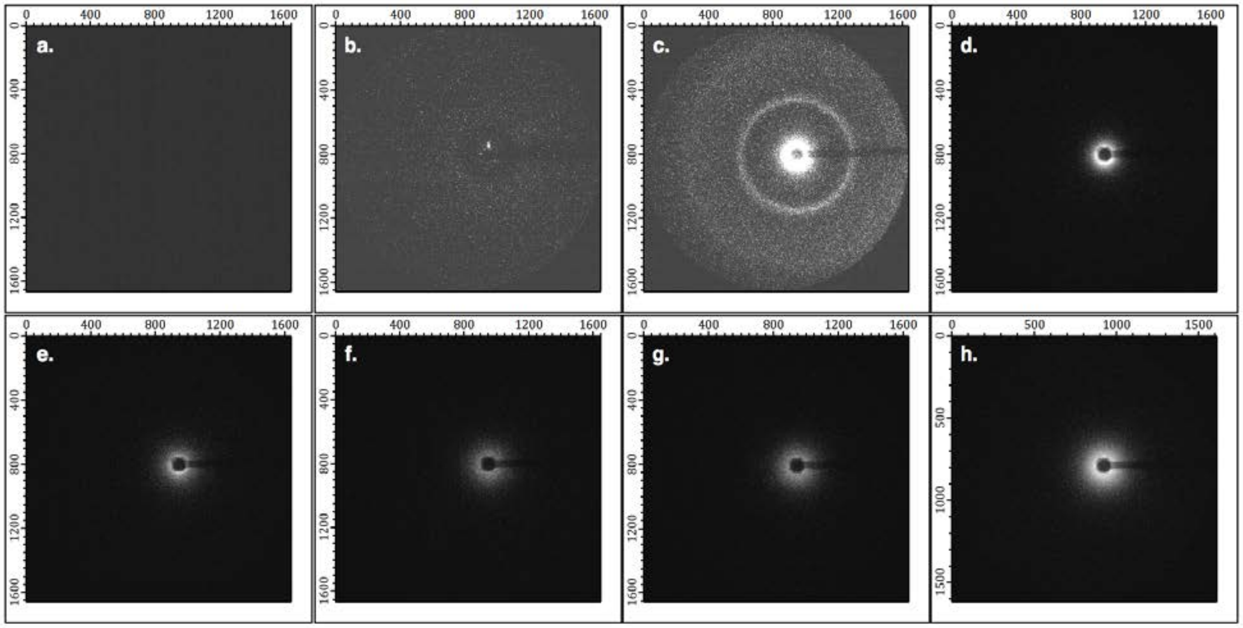

Supplementary Figure 8. Raw images obtained from the experiment shown in Figure 2a from campaign 1: (a) the dark field (no X-rays), (b), empty chamber, (c) Ag behenate (for calibration), (d) Static DNTF, (e) 0.06 μs, (f) 0.21 μs, (g) 0.36 μs, (h) 0.52 μs. The gray scale for images in (a – c) is log-spaced from 500 (black) to 1000 (white). The gray scale for images in (d – g) is log-spaced from 500 (black) and 7000 (white).

As there is an unknown amount of background scattering in experimental SAXS data, $I^*_{dp}(q)$, we need to be able to determine any parameter sensitivity to the choice of Tr*; specifically, $\Delta\rho$. For a given value of Tr, $I^*_{dp}(q)$ can be modeled from the ideal $I_{dp}(q)$ by the equation:

(12)
$$I^*{}_{dp}(q) = \frac{\mathrm{Tr}}{\mathrm{Tr}^*} I_{dp} + \frac{C}{t^*} I_b(q) \left[\frac{\mathrm{Tr}}{\mathrm{Tr}^*} - 1\right]$$

where $I_{dp}(q)$ is the actual SAXS data that is used with the actual transmission, Tr, and the estimated transmission, Tr*. Supplementary Equation 12 is derived using Supplementary Equation 11 for the $I_{dp}(q)$ and $I^*_{dp}(q)$, followed by substitution of $I_{raw}(q)$. Therefore, the uncertainty of Tr can be accounted for, as well as its effect on the model fit parameters. Experimentally, the scale of $I_{raw}(q)$ increases post detonation such that $I_{raw}(q) >> I_b(q)$ and therefore Tr does not change the shape of the $I_{dp}(q)$ at later times. At early times, Tr is expected to be very near Tr^*. Nevertheless, Supplementary Equation 12 is used to demonstrate that the uncertainty in Tr does not affect the trend in $\Delta\rho$, from which the transition from liquid to onion is elucidated from the SAXS data (see section 1, above). In general, the incorporation of background scattering does decrease the contrast of the layers and increases the error for the SAXS data collected at 1 μs. However, the presence of an onion-like morphology remains with good certainty.

In these experiments, an unconfined, cylindrical HE, is initiated by a much smaller EFI. Consequently, the detonation front exhibits radially symmetric curvature, with the edges of the HE detonating later than the

center axis of the cylinder. The X-rays transit the transverse axis and therefore the probed volume can contain both detonated and undetonated signals. This is observed within the first ~ 65 ns post-detonation for DNTF and further confirmed by thermochemical modeling, assuming a DNTF detonation front velocity. The relative amount of undetonated HE is determined to be the amount that results in constant positive SAXS at $q < 0.02$ Å^{-1}. The uncertainty in the relative amount of undetonated DNTF scatters at low-q and primarily affects the size distribution of onions.

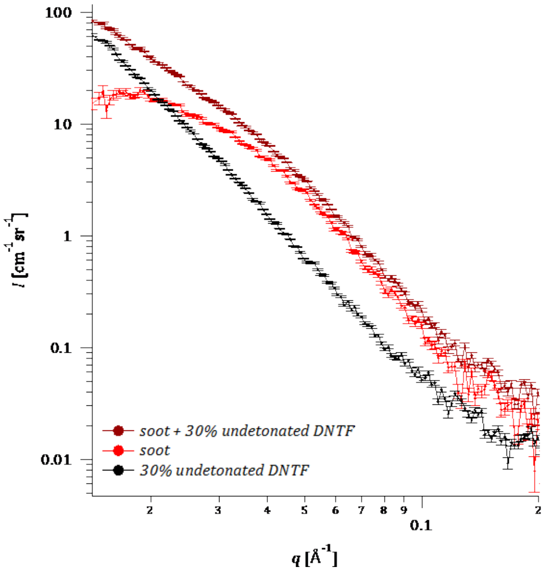

Supplementary Figure 9. Comparison of TR-SAX data from DNTF collected at 0.09 μs after detonation (dark red), to recovered detonation product (red) and 30% undetonated DNTF (black). Error bars are calculated using the standard error of mean (Methods).

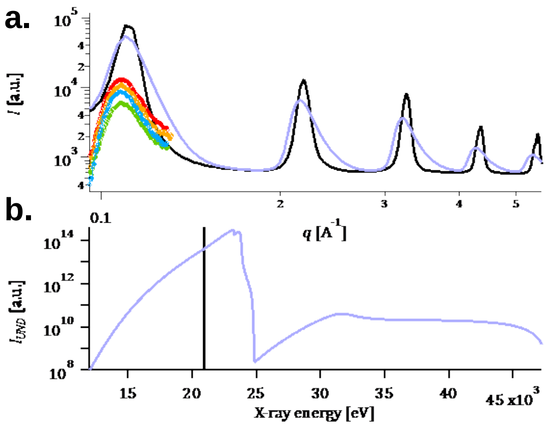

Supplementary Figure 10. (a) Silver behenate SAXS trace collected from a monochromatic X-ray beam at 9-IDC (black) with the expected scattering from the U18 with Rh mirrors (blue), and the measured scattering from the U18 with Rh mirror from the four different cameras (circles). The expected U18 undulator profile is shown in (b). The energy spectrum is similar for the U17.2 undulator used in the 2nd experimental campaign.

The absolute intensity was obtained using a 2 mm thick piece of glassy carbon. The absolute scattering cross-section from this glassy carbon was obtained from the USAXS beamline at 9-IDC at the advanced photon source.[6] The calibration factor, *C*, for each camera was obtained by matching the *q*-independent intensity from the scattering cross-section to the SAXS pattern obtained with the TR-SAXS detector. We note that wavelength smearing does affect the shape of the SAXS curve for glass carbon but not the absolute scale.

The *q*-calibration is an essential part of any SAS measurement and allows for an accurate reporting of scattering sizes. Here, the *q* calibration was determined using a silver behenate calibration standard[7]. With this standard, the sample to detector distance and beam center for each camera were obtained using the Nika package[8] for Igor Pro. Within this routine, a peak function is fit to the data and extracts the peak position and compares to it to the expected peak position that is based on the *d*-spacing for silver behenate.[7] The absolute intensity calibration was accomplished by measuring the SAXS from 2 mm thick glassy carbon.[9] The absolute scattering cross-section from glassy carbon was obtained from 9-IDC, using monochromatic X-rays and is comparable with the TR-SAXS data.
The *q*-calibration above is based on the monochromatic scattering from silver behenate. The polychromaticity is accounted for by smearing a model (Supplementary Equation 8) using a $\langle\lambda\rangle$ value that aligns the peak positions from polychromatic and monochromatic silver behenate scattering. To illustrate the effect of wavelength smearing on the calibration, the SAXS from silver behenate was obtained from both monochromatic and polychromatic X-rays and are shown in Supplementary Figure 10. Using Supplementary Equation 8, the monochromatic data was smeared, using the $R(q,\langle q\rangle)$ profile for silver behenate, and compared with the measured polychromatic data. From Supplementary Figure 10, the smearing causes some asymmetry in the diffraction peaks observed in both the experimental and theoretical data.

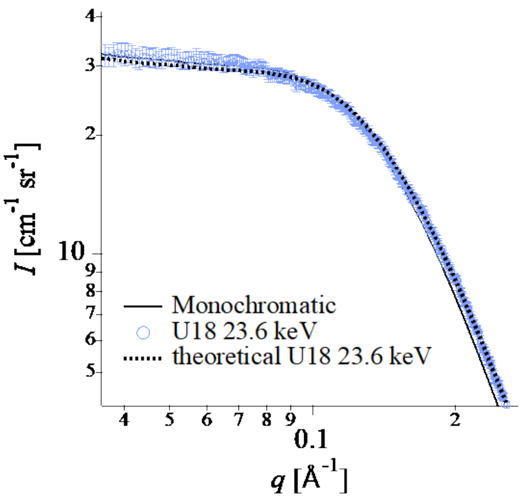

Supplementary Figure 11. Scattered intensity obtained from glassy carbon using monochromatic X-rays (black line) and pink-beam X-rays (blue). The theoretical pink-beam scattering from the glassy carbon is shown as a dashed line. Error bars are calculated using the standard error of mean (Methods).

The absolute intensity calibration is necessary for our experiments for three reasons: (1) the intensity scaling for each camera is different, (2) the absolute scattering cross-section allows for an estimation of the volume fraction or contrast that is used in the model fitting, and (3) provide additional verification of Supplementary Equation 8 so that it can be applied in the model fitting. All of the above requirements are satisfied using 2 mm thick glassy carbon, which is a common absolute calibration standard.[9] The absolute scattering cross-section from glassy carbon was obtained from 9-IDC, using

monochromatic X-rays. The SAXS from the same sample was collected from the 4-camera system. The 1-D $I(q)$ curve from each camera was compared with the calibrated data to obtain the calibration factor, C, for each camera. The intensity matching method used to obtain C is applied to the q-range: $0.04\ \text{A}^{-1} < q < 0.1\ \text{A}^{-1}$; the $I(q)$ curve at higher q values has a different shape and cannot be used for this purpose. The slight change in shape can be accounted for by applying Supplementary Equation 8 to the monochromatic data and shown in Supplementary Figure 11. As the scattering from detonation soot is expected to be more similar to glassy carbon than Ag behenate, we expect that the effect polychromaticity to be minimal. Nevertheless, it is included for completeness.

In Composition B, a high fraction of nano-diamond could be discerned, with typical sizes ranging between 5 and 7 nm. Composition B is well-known to produce an abundance of nano-diamond with morphologies consistent with these observations[10]. The mean size[11, 12, 13] and fine-structure[13, 14] can be heuristically extracted from the TR-SAXS data using a two-level unified equation to represent the nano-diamond and structure factor that accounts for the mass-fractal-like scattering from the aggregate structure; a more complete treatment of nano-diamond from Composition B is the subject of a future publication. The purpose of the modeling here is to extract the mean particle size and fine structure from the TR-SAXS and compare it with HNS. The DND model, $I_{\text{DND}}(q)$, simulates the SAXS from a monodisperse population of particles with some fine-structure by the equations:

(13)
$$I_{\text{DND}}(q) = S_{\text{f}}(d_{\text{m}}) I_{\text{D}}(q) + I_{\text{FS}}(q) + b$$

(14)
$$I_{\text{D}}(q) = \mathrm{G_D}\, \mathrm{e}\left(\frac{-q^2 R_{\text{D}}^2}{3}\right) + \mathrm{B(R_D, G_D)} \left[\frac{\left[\text{erf}\left(\frac{qR_{\text{D}}}{\sqrt{6}}\right)\right]^3}{q}\right]^4$$

(15)
$$I_{\text{FS}}(q) = \mathrm{G_f}\, \mathrm{e}\left(\frac{-q^2 R_{\text{f}}^2}{3}\right) + \mathrm{B(R_f, G_f)} \left[\frac{\left[\text{erf}\left(\frac{qR_{\text{f}}}{\sqrt{6}}\right)\right]^3}{q}\right]^4$$

(16)
$$\mathrm{B(R_i, G_i)} = \frac{G_{\text{i}} 4.86}{R_{\text{i}}^{\;4}}$$

where R_{D}, G_{DND} are the radius of gyration and scattering power of the nano-diamond, R_{f}, G_{f} *are the* radius of gyration and scattering power of the nano-diamond fine structure that appear in the high-q region, $S_{\text{f}}(d_{\text{m}})$ is the structure factor for a mass fractal aggregate[15] with a fractal dimension, d_{m}, and termination radius equal to the nano-diamond radius $\left(R_{\text{D}}\sqrt{5/3}\right)$ and b is the flat background. $B(R_{\text{i}},G_{\text{i}})$ is a scaling factor associated with power law scattering that assumes a log-normal size-distribution of spheres[16] with a standard deviation of 0.3, which is consistent with TEM imaging. Using Supplementary Equation 16 allows for the presence of a size-distribution[16] and reduces the number of fit parameters. The mass-fractal scattering at low-q could be modeled by another unified level but would introduce another fit parameter. Therefore, the structure factor[15] for a mass fractal aggregate is used. In total, there are 6 fit parameters that

were fit using least-squares fitting via Supplementary Equation 2. From the least-squares fit of Supplementary Equation 13, the mean diameter of the nano-diamond is 7 nm and contains smaller heterogeneities on the order of 1 – 2 nm. The model fits for data collected at two different times are shown in Supplementary Figure 12.

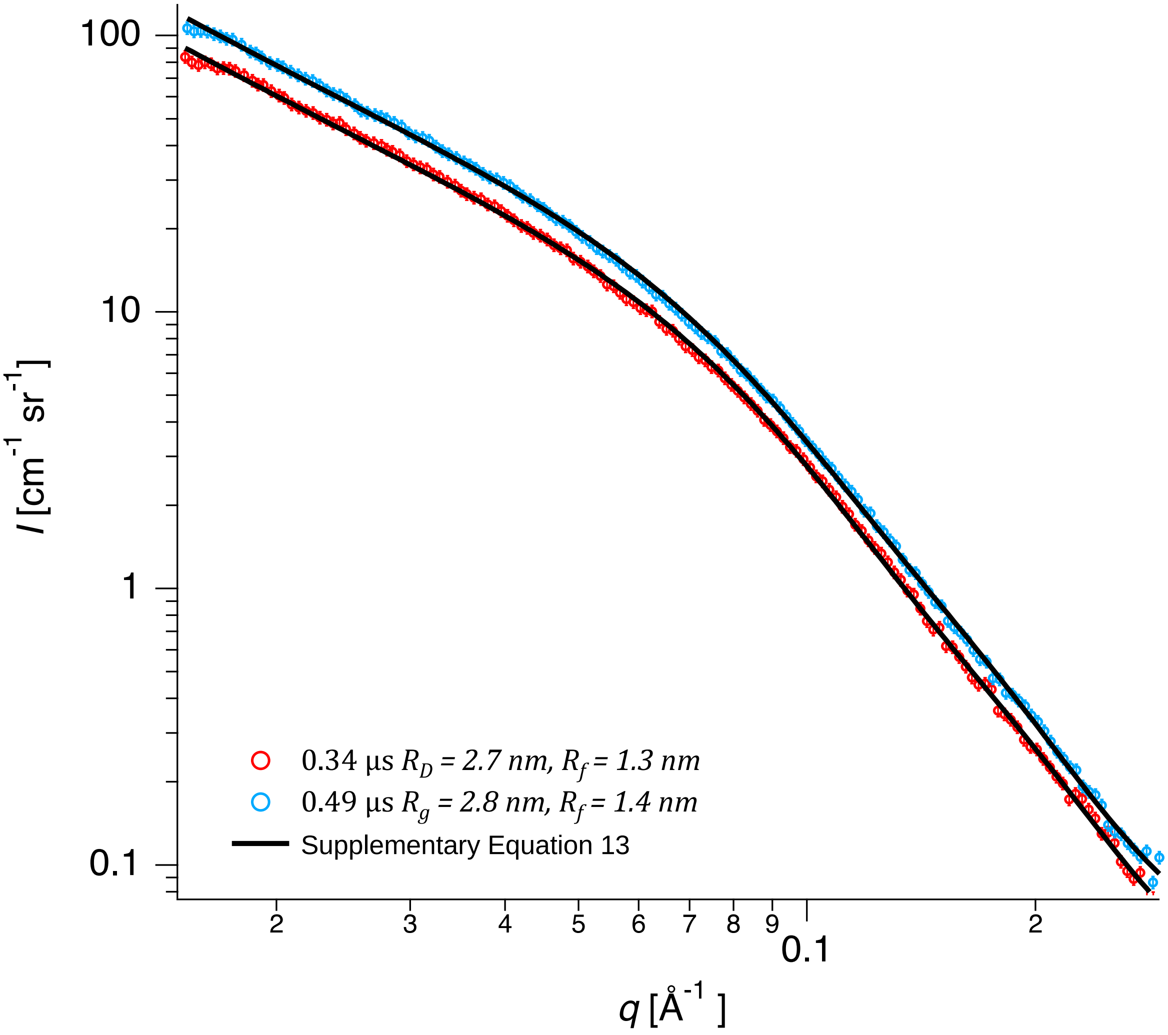

Supplementary Figure 12. SAXS data collected from Comp B detonation with the model fits of Supplementary Equation 13. Errors in the radii of gyration were 0.1 nm. Error bars are calculated using the standard error of mean (Methods).

The recovered carbonaceous detonation products from HNS contain a mixture of graphitic ribbons and nano-diamond, with the former being more abundant as evidenced by TEM imaging and TR-SAXS. Therefore, the TR-SAXS data obtained from HNS was fit to a single level unified equation, which combines the Guinier approximation with a power-law behavior[17]; the latter can be attributed to a number of different morphologies, depending on the exponent. A single level unified equation is given by:

(17)

$$I_{\mathrm{HNS}}(q) = \left\{ \mathrm{G_r}\, \mathrm{e}\left(\frac{-q^2 R_{\mathrm{r}}^2}{3}\right) + \mathrm{B_r}\left[\frac{\left[\mathrm{erf}\left(\frac{q R_{\mathrm{r}}}{\sqrt{6}}\right)\right]^3}{q}\right]^{\mathrm{P_r}} \right\}$$

where G_r and B_r are scaling factors, R_r is the radius of gyration of the soot, P_r is a power-law exponent (=4 for a Porod Decay). An exponent slightly greater than 3 is obtained from the fit of Supplementary Equation 17, which suggests a convoluted structure with heterogeneities within[18] the phase and/or at the surface[19]. The model fits are shown in Supplementary Figure 13. Because P_r is less than four and there is no apparent Guinier knee associated with the size of the fine-structure, the carbonaceous detonation products formed in HNS contain fine-structure that is too small to be resolved our measurements.

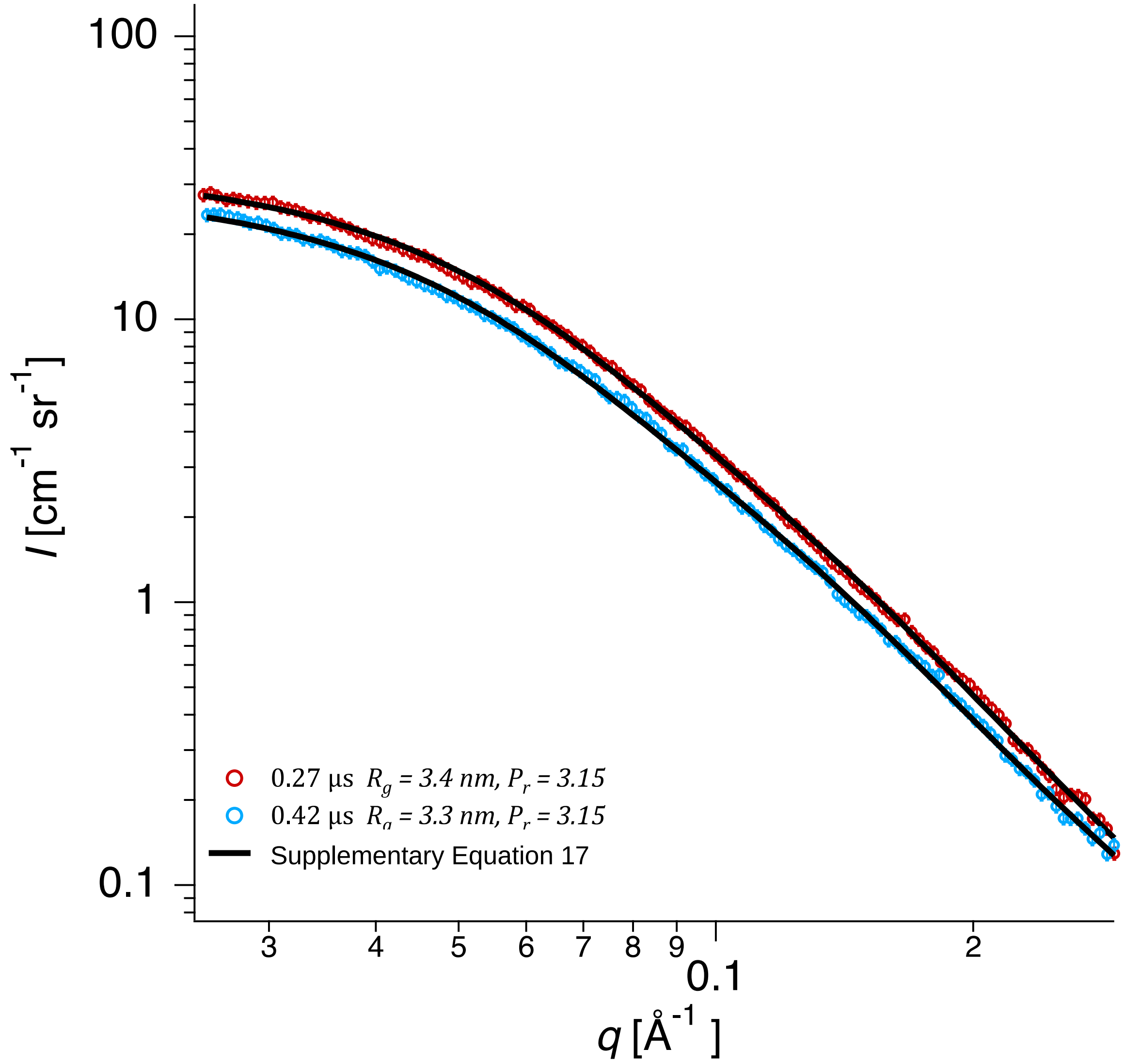

Supplementary Figure 13. SAXS data collected from HNS detonation (circles) with the model fit of Supplementary Equation 17. Errors in the radii of gyration were 0.1 nm. Error bars are calculated using the standard error of mean (Methods).

Supplementary References:

1. Guinier A, Fournet G. *Small-angle scattering of X-rays*. Wiley (1955).

2. Glatter O, Kratky O. *Small Angle X-Ray Scattering*. Academic Press (1982).

3. Newville M, Stensitzki T, Allen DB, Ingargiola A. LMFIT: Non-Linear Least-Square Minimization and Curve-Fitting for Python. https://lmfit.github.io/lmfit-py/

4. Dorenbos P, Haas JTMd, Eijk CWEv, Melcher CL, Schweitzer JS. Nonlinear response in the scintillation yield of Lu2SiO5:Ce3+. *IEEE Transactions on Nuclear Science* **41**, 735-737 (1994).

5. Bagge-Hansen M*, et al.* Measurement of carbon condensates using small-angle x-ray scattering during detonation of the high explosive hexanitrostilbene. *J Appl Phys* **117**, (2015).

6. Ilavsky J, Allen AJ, Levine LE, Zhang F, Jemian PR, Long GG. High-energy ultra-small-angle X-ray scattering instrument at the Advanced Photon Source. *J Appl Crystallogr* **45**, 1318-1320 (2012).

7. Gilles R, Keiderling U, Strunz P, Wiedenmann A, Fuess H. Silver Behenate as a Standard for Instrumental Resolution and Wavelength Calibration for Small Angel Neutron Scattering. Materials Science Forum **321-324**, 264-269 (2000).

8. Ilavsky J. Nika: software for two-dimensional data reduction. *J Appl Crystallogr* **45**, 324 – 328 (2012).

9. Zhang F, Ilavsky J, Long GG, Quintana JPG, Allen AJ, Jemian PR. Glassy Carbon as an Absolute Intensity Calibration Standard for Small-Angle Scattering. *Metallurgical and Materials Transactions A-physical Metallurgy and Materials Science* **41A**, 1151 – 1158 (2010).

10. Danilenko VV. On the history of the discovery of nanodiamond synthesis. *Phys Solid State* **46**, 595-599 (2004).

11. Pichot V, Risse B, Schnell F, Mory J, Spitzer D. Understanding ultrafine nanodiamond formation using nanostructured explosives. *Scientific Reports* **3**, (2013).

12. Avdeev MV, Tomchuk OV, Ivankov OI, Alexenskii AE, Dideikin AT, Vul AY. On the structure of concentrated detonation nanodiamond hydrosols with a positive zeta potential: Analysis of small-angle neutron scattering. *Chem Phys Lett* **658**, 58-62 (2016).

13. Mykhaylyk OO, Solonin YM, Batchelder DN, Brydson R. Transformation of nanodiamond into carbon onions: A comparative study by high-resolution transmission electron microscopy, electron energy-loss spectroscopy, x-ray diffraction, small-angle x-ray scattering, and ultraviolet Raman spectroscopy. *J Appl Phys* **97**, (2005).

14. Pichot V, Comet M, Risse B, Spitzer D. Detonation of nanosized explosive: New mechanistic model for nanodiamond formation. *Diamond Relat Mater* **54**, 59-63 (2015).

15. Teixeira J. Small-angle Scattering By Fractal Systems. *J Appl Crystallogr* **21**, 781 – 785 (1988).

16. Beaucage G, Kammler HK, Pratsinis SE. Particle size distributions from small-angle scattering using global scattering functions. *J Appl Crystallogr* **37**, 523-535 (2004).

17. Beaucage G. Approximations leading to a unified exponential power-law approach to small-angle scattering. *J Appl Crystallogr* **28**, 717-728 (1995).

18. Martin JE, Hurd AJ. Scattering from Fractals. *J Appl Crystallogr* **20**, 61-78 (1987).

19. Bale HD, Schmidt PW. SMALL-ANGLE X-RAY-SCATTERING INVESTIGATION OF SUBMICROSCOPIC POROSITY WITH FRACTAL PROPERTIES. *Phys Rev Lett* **53**, 596-599 (1984).
